# Role of GABA_A_ receptors in alcohol use disorders suggested by chronic intermittent ethanol (CIE) rodent model

**DOI:** 10.1186/s13041-017-0325-8

**Published:** 2017-09-20

**Authors:** Richard W. Olsen, Jing Liang

**Affiliations:** 1 Department of Molecular and Medical Pharmacology, David Geffen School of Medicine at UCLA, Los Angeles, CA 90095 USA; 2 Titus Family Department of Clinical Pharmacy, School of Pharmacy, University of Southern California, Los Angeles, CA 90089 USA

**Keywords:** GABA_A_ receptors, Rodent model of alcoholism, Chronic intermittent ethanol, Inhibitory synaptic plasticity

## Abstract

GABAergic inhibitory transmission is involved in the acute and chronic effects of ethanol on the brain and behavior. One-dose ethanol exposure induces transient plastic changes in GABA_A_ receptor subunit levels, composition, and regional and subcellular localization. Rapid down-regulation of early responder δ subunit-containing GABA_A_ receptor subtypes mediating ethanol-sensitive tonic inhibitory currents in critical neuronal circuits corresponds to rapid tolerance to ethanol’s behavioral responses. Slightly slower, α1 subunit-containing GABA_A_ receptor subtypes mediating ethanol-insensitive synaptic inhibition are down-regulated, corresponding to tolerance to additional ethanol behaviors plus cross-tolerance to other GABAergic drugs including benzodiazepines, anesthetics, and neurosteroids, especially sedative-hypnotic effects. Compensatory up-regulation of synaptically localized α4 and α2 subunit-containing GABA_A_ receptor subtypes, mediating ethanol-sensitive synaptic inhibitory currents follow, but exhibit altered physio-pharmacology, seizure susceptibility, hyperexcitability, anxiety, and tolerance to GABAergic positive allosteric modulators, corresponding to heightened alcohol withdrawal syndrome. All these changes (behavioral, physiological, and biochemical) induced by ethanol administration are transient and return to normal in a few days. After chronic intermittent ethanol (CIE) treatment the same changes are observed but they become persistent after 30 or more doses, lasting for at least 120 days in the rat, and probably for life. We conclude that the ethanol-induced changes in GABA_A_ receptors represent aberrant plasticity contributing critically to ethanol dependence and increased voluntary consumption. We suggest that the craving, drug-seeking, and increased consumption in the rat model are tied to ethanol-induced plastic changes in GABA_A_ receptors, importantly the development of ethanol-sensitive synaptic GABA_A_ receptor-mediating inhibitory currents that participate in maintained positive reward actions of ethanol on critical neuronal circuits. These probably disinhibit nerve endings of inhibitory GABAergic neurons on dopamine reward circuit cells, and limbic system circuits mediating anxiolysis in hippocampus and amygdala. We further suggest that the GABA_A_ receptors contributing to alcohol dependence in the rat and presumably in human alcohol use disorders (AUD) are the ethanol-induced up-regulated subtypes containing α4 and most importantly α2 subunits. These mediate critical aspects of the positive reinforcement of ethanol in the dependent chronic user while alleviating heightened withdrawal symptoms experienced whenever ethanol is absent. The speculative conclusions based on firm observations are readily testable.

## Background

### Definition of alcohol use disorders

Alcohol use disorders (AUD) are defined as alcohol abuse and alcohol dependence clinically defined as drinking—or being sick from drinking—that interferes with taking care of one’s home or family, or causes job troubles, or school problems, creating large problems both for society and for the drinkers themselves [1, 2]. AUD represent a substantial public health problem worldwide. According to the World Health Organization (WHO) 2015 report, the harmful use of alcohol results in approximately 3.3 million deaths per year world-wide [3]. Approximately 7.2% or 17 million adults in the United States ages 18 and older had an AUD in 2012. This includes 11.2 million men and 5.7 million women. Adolescents can be diagnosed with an AUD as well, and in 2012, an estimated 855,000 adolescents ages 12–17 had an AUD [2, 4].

### Molecular actions of ethanol on the brain, GABA_A_ receptors, and other potential ion channel targets, development of drug dependence after chronic ethanol

GABA_A_ receptors (GABA_A_Rs) have long been implicated in mediating at least part of the actions of ethanol (EtOH) in mammalian brain. The molecular mechanism(s) of action for intoxicating doses of EtOH have been especially of interest even before the advent of the Research Society on Alcoholism. In recent years, however, the focus of EtOH research has shifted to identifying a protein receptor-based target for EtOH, and several ligand-gated ion channels (LGIC), which include NMDA- [5] and non-NMDA-type glutamate receptors [6, 7], serotonin 5-HT3 receptors [8], inhibitory glycine receptors, purinergic receptors (P2X) [9, 10], and GABA_A_Rs [11–13], as well as voltage-gated ion channels (VGIC): G-protein coupled inwardly rectifying K^+^ channels (GIRK) [14], and Big Potassium (BK) channels, have been implicated in ethanol’s actions on the brain. Whether EtOH acts directly or indirectly on these membrane channel proteins is not totally established. EtOH is accepted to have a GABA-mimetic effect. However, some important effects of EtOH on GABA_A_R-mediated inhibition may be presynaptic [15–18]. Nevertheless, in either case (presynaptic or postsynaptic action on GABAergic transmission), considerable evidence favors the direct action on specific protein targets. We believe there is strong evidence for direct action on some channels, especially GABA_A_Rs. In this mini-review we give a brief review of evidence suggesting GABA_A_Rs involvement in AUD, with a detailed summary of the Chronic Intermittent Ethanol (CIE) rodent model, emphasizing studies in our lab.

To date, the mechanisms for how excess EtOH consumption leads to alterations in the human brain that produce alcohol dependence remain murky. The formation of AUD is a chronic and complex process. EtOH affects brain function by interacting with multiple neurotransmitter systems, especially disruption of the delicate balance between GABA, the primary inhibitory neurotransmitter, and glutamate, the major excitatory neurotransmitter in the central nervous system (CNS) [19]. Short-term alcohol exposure tilts this balance toward CNS depression, while under long-term alcohol exposure, the brain attempts to compensate by bringing the balance back toward equilibrium. These neurobiological changes present behaviorally as the development of tolerance to EtOH’s sedative effects. When EtOH consumption is abruptly discontinued or reduced, these compensatory changes are no longer opposed by the presence of EtOH, thus leading to the excitation of neurotransmitter systems and the development of the alcohol withdrawal syndrome (AWS) [20].

### Evidence suggesting GABA_A_Rs involvement in AUD

Several lines of evidence suggest a possible role of GABA_A_Rs in AUD. Here is a list of some of these; space does not allow a thorough review of these subjects nor a thorough evaluation of the pros and cons for the theoretical connection, but present some examples.

1. Human genetic link of AUD and GABA_A_Rs.
2. Plasticity of neurotransmission triggered by experience (learning and memory), including exposure to neuroactive drugs, with development of dependence. Concept of over-stimulation by agonists or positive allosteric modulators inducing down-regulation of target receptors and compensatory additional receptor changes.
3. Acute EtOH and GABA_A_Rs. Direct action on GABA_A_R subtypes.
4. Effects of in vivo chronic administration of EtOH: internalization of GABA_A_R subtypes and altered subunit gene expression, subtype cell surface levels, and localization involving trafficking.
5. Correspondence of time course of EtOH-induced plastic changes in GABA_A_R subtypes with behavioral alterations associated with EtOH withdrawal and dependence development. The CIE rodent model of alcohol dependence.

1). Genetics.

AUD is a complicated behavioral disorder with complex genetic involvement. Genes encoding a cluster of GABA_A_R subunits *GABRA4, GABRA2, GABRB1, and GABRG1* on chromosome 4 are associated with certain aspects of alcoholism in humans. Gene clusters are well known to exhibit co-regulation of expression. There are several GABA_A_R subunit gene clusters, and some have been reported to show developmentally controlled co-expression of the gene products [21], suggesting some combination of these proteins acting together functionally in some way, presumably the heteropentameric α4βγ or α2β1γ1 subtypes, could affect alcohol behavior. Single nucleotide polymorphisms (SNPs) in the chromosome 4 GABA_A_R subunit genes are highly associated with alcohol abuse and dependence [22–24]. In fact the important α2 subunit [25] shows the highest association with AUD of any gene in the human genome [26]. Why these genes show behavioral association is not clear, but some animal evidence suggests that the α2 subunit-containing GABA_A_Rs participate functionally in critical neurocircuitry involved in the positive reinforcing effects of EtOH including anxiolysis [27–30] (discussed below), as they are for benzodiazepines (BZs) [31–34], and other drugs of abuse, such as cocaine [35]. We posit that the α2-GABA_A_Rs are needed for the development of EtOH dependence, with evidence below. Increased expression and function of these GABA_A_Rs might be associated with dependence, and reduced expression and function somehow associated with less susceptibility to developing dependence. Note that both the α4 [36] and δ [37] GABA_A_R subunits in ventral striatum (nucleus accumbens in dopamine reward circuit) are also required for high levels of voluntary EtOH consumption (Commentary [38]).

2). Plasticity of neurotransmission triggered by experience (learning and memory), including exposure to neuroactive drugs, and development of dependence.

Synaptic plasticity is most often described, to be as simplistic as possible, as a strengthening or weakening of synaptic strength in response to activating that synapse. This is probably best typified by the phenomenon of long-term potentiation (LTP) in the hippocampus as a synaptic model of memory [39]. In this model, tetanic (100 Hz for 1 s) stimulation of the perforant pathway input to hippocampal field CA1 results in LTP of excitatory synapses and plastic changes in the synaptic AMPA- and NMDA-type glutamate receptors, changing their expression levels, or subunit composition, and/or localization [40]. The mechanisms proposed for producing synaptic plasticity are many, involving either presynaptic or postsynaptic changes or both [41]. The suggested postsynaptic mechanisms involve protein phosphorylation-controlled membrane insertion, removal, rearrangement of receptors, or mysterious change in receptor conductance [42, 43]. This is usually but not always considered a use-dependent synaptic strengthening. On the other hand, use-dependent down-regulation of neurotransmitter receptors is a well-described phenomenon [44, 45]. The ratio of excitation to inhibition is regarded as so important that a new concept called *scaling* has been put forward (e.g., [46]), in which compensatory changes in excitation or inhibition accompany any perturbation of the other (inhibition or excitation). Nevertheless, examples abound in which the deciding factor for aberrant plasticity is reduced GABAergic inhibitory function, which seems particularly susceptible to derangement. These examples cover several chronic drug models as well as epilepsy. Application of GABAergic positive allosteric modulator (PAM) drugs, or even GABA itself, to the mammalian cerebral cortex produces withdrawal signs upon removal, such that even an hour exposure can produce long-lasting focal seizures upon termination, the so-called “GABA withdrawal syndrome” [47–49] and that modified GABA_A_Rs are found in many types of human and experimental epilepsy [50–54]. Status epilepticus induces massive synaptic release of GABA and protein phosphorylation-dependent down-regulation of synaptic GABA_A_Rs [55, 56], leading to plastic changes in other GABA_A_R subtypes including extrasynaptic ones [57]. Likewise, administration of, and in some cases withdrawal from, any GABA_A_R PAM drug, including neurosteroids [58], BZs [59, 60], and anesthetics [61] can induce GABA_A_R down-regulation, compensatory plasticity, producing tolerance and withdrawal and aberrant plasticity involving GABA_A_Rs and associated negative effects on behaviors. We present evidence that EtOH is also a PAM with this potential for harm via chronic over-stimulation-induced aberrant plasticity, and in fact, involvement in AUD.

3). Acute EtOH and GABA_A_Rs.

Single or acute alcohol consumption is an alcohol intake that occurs over a short period of time.

The effects of single alcohol consumption depend on alcohol concentration and the amount of intake. EtOH concentrations in the brain can vary in a range from a few millimolar after one drink to more than 100 millimolar, which induces sleep in a naïve individual. As a CNS depressant, EtOH in a concentration range of ≥5 ~ 10 mM (about 3 drinks) leads first to a feeling of being ‘high’ or ‘buzzed’: mood elevation, talkativeness, increased socialization, disinhibition of shyness, and grandiose thoughts, followed by sedation accompanied by decreased attention, impaired decision making, impaired coordination/ locomotion, alterations in memory, mood changes, and lethargy [15]. These behavioral changes are accompanied by an apparent increase in GABA_A_R inhibition and decreased glutamatergic excitation [62, 63]. The legal limit for driving a car in the USA is 0.08% [64], about 17 mM in serum and something similar in brain CSF [65]. A large number of animal experiments have shown EtOH effects on the brain. EtOH is accepted to have a GABA-mimetic effect, and an acute anxiolytic effect, which is at least in part related to the potentiation of GABAergic neurotransmission in the basolateral amygdala (BLA) [66]. However, as with the case of glutamate receptor synaptic plasticity in LTP [41], and in alcohol actions [5], in addition to postsynaptic GABA_A_R interactions of EtOH [11, 67, 68], some important effects of EtOH on GABA_A_R-mediated inhibition may be presynaptic [16, 17]. In vitro studies on neurons in brain slices, or in culture, or even brain membrane homogenates, demonstrate that application of EtOH at 20 ~ 100 mM stimulates GABA-activated Cl^−^ channels (GABA_A_Rs: [69, 70]). In studies of effects on neurons using patch clamp recordings in slices prepared after intraperitoneal injection in rats of EtOH (3 g/kg), a rapid down-regulation of GABA_A_R phasic and tonic inhibitory currents was observed in hippocampus within 5 ~ 15 min. This change was accompanied by plastic changes in GABA_A_R subunit cell surface levels and localization consistent with a net subunit switch [65, 71]. These effects of acute EtOH exposure on GABA_A_Rs are transient and reversible; understanding the process of GABA_A_Rs interacting with EtOH from the time of exposure to recovery can provide valuable information for how dependence develops with long term EtOH exposure.

Several lines of evidence support direct action of EtOH on GABA_A_Rs. GABA_A_Rs have been implicated in mediating the anxiolytic, mood-enhancing, and motor incoordination effects of alcohol at blood alcohol levels of 10 ~ 30 mM [11, 62, 68, 70, 72, 73]. GABA_A_R antagonists reduce EtOH effects in vivo, while agonists and PAMS enhance EtOH effects [62]. Systemic EtOH enhances GABA_A_R-mediated inhibition of target cells but does not show much direct action on such cells [74, 75]. Enhancement of GABA_A_R synapses is widely observed (e.g., [76]) but some reports noted that these EtOH actions on GABA_A_R synapses could be presynaptic [16, 17]. Others demonstrated direct enhancement of GABA_A_R function by EtOH in the assay in brain membane homogenates containing synaptoneurosomes [77, 78] and in primary cultured neurons [79]. In neurons recorded from brain slices, α4/6βδ GABA_A_R subtype-mediated tonic inhibitory currents are uniquely sensitive to alcohol (≤30 mM) EtOH concentrations [80–84]. High EtOH sensitivity (≤10 mM) has also been reported in recombinantly expressed α4/6βδ receptors [85], with significant β3 selectivity [86]. Other workers (e.g., Borghese et al., [87]), did not see EtOH effects on GABA_A_R currents. Clearly they are region and cell-type specific and of variable amplitude.

4). Effects of in vivo chronic administration of EtOH: internalization of GABA_A_R subtypes, altered subunit gene expression, subtype cell surface levels, and trafficking/subcellular localization.

The finding of EtOH-induced GABA_A_R plasticity was based on earlier observations on ionotropic glutamate receptors [43] and actions of BZs on GABA_A_Rs [88]. Chronic administration of BZs leads to tolerance to the traditional ‘agonist’ effects of diazepam. The effect of chronic agonist BZs on GABA_A_R modulation by BZs was at first interpreted as ‘uncoupling’ of receptors for GABA and BZs [89]. However, Gallager and colleagues [90] used implanted dialysis tubing to administer diazepam continuously for many days and observed reduction in GABA_A_R-mediated transmission in several brain areas, *not* just reduction of BZ modulation of GABA_A_R synapses. Poisbeau et al. [91] pointed out the ‘silencing’ of GABA_A_R synapses in some regions of hippocampus during flurazepam withdrawal. Primus et al. [92] demonstrated uncoupling of BZ modulation of GABA binding to GABA_A_Rs in membrane homogenates after exposure of recombinant cells expressing GABA_A_Rs to one hour or more of BZs prior to homogenization. But, this was explained by our observation [93] that the enhancement of BZ binding produced by GABA was retained by the receptor protein after BZ treatment of the cells, because the homogenization resulted in membrane vesicles (endosomes) which exhibited BZ binding inside the vesicles that was insensitive to GABA which could not penetrate the membranes to reach the receptor binding sites, whereas the radioactive BZ could enter. Brief treatment of the membrane vesicles with osmotic shock, centrifugation, and resuspension in fresh assay buffer allowed detection of the same number of GABA_A_R-BZ binding sites with intact GABA enhancement. This was consistent with the receptor being internalized and no longer functioning at the cell surface, i.e., down-regulation of GABA_A_R proteins that are sensitive to a given BZ after over-stimulation by that BZ.

However, tolerance to diazepam and its congeners was accompanied by an increase in efficacy for inverse agonists; further, chronic administration of inverse agonists and lead to ‘chemical kindling’ of seizures [94, 95]. This led to a teeter-totter model of receptor plasticity, suggested as a change in set point of intrinsic activity for allosteric modulatory drugs, with unknown structural explanation [94]. This idea was supported by the observation of [96] that tolerance to chronic diazepam was reduced persistently after a single exposure to the BZ antagonist flumazenil. However, others [97] showed that kindling by a negative allosteric modulator (NAM) β-carboline could co-exist with diazepam tolerance in mice treated chronically, suggesting the two events are independent. Nevertheless, there was more new information in addition to the evidence by Gallager and colleagues and many others including us for loss of GABA_A_Rs and not just BZ modulation, apparently due to internalization of BZ-modulated GABA_A_Rs [71, 98, 99]. Importantly, the cloning of a family of GABA_A_R subunit genes and demonstration of a family of heteropentameric receptor subtypes differing in localization as well as pharmacology and regulatory mechanisms would appear to account for a complicated tolerance pattern for BZs of differing chemical structure [89].

Ticku and colleagues [62] showed EtOH-induced plasticity of GABA_A_Rs including functional reduction of GABA_A_R-mediated transmission, and increased efficacy for excitatory inverse agonists like Ro-15-4513 (partial inverse agonist) and β-carbolines, accompanied by an up-regulation of diazepam-insensitive (DZ-IS) binding of [^3^H]Ro15–4513 in forebrain and cerebellum [100], shown later to be due to α4 and α6 subunits, respectively. This is homologous to observations with other GABAergic drugs like BZs. A similar effect of chronic EtOH exposure (CIE [67]) is apparent, producing down-regulation of EtOH- (and diazepam-) sensitivity of GABA_A_Rs, but increased sensitivity to inverse agonist BZ-site NAMs, explained by EtOH-induced GABA_A_R plasticity.

Similar increases in GABA_A_R α4 subunit and smaller changes in some other subunits were observed by several groups in rodents treated with chronic EtOH, e.g., Ticku and colleagues [101, 102]; and Morrow and colleagues [103, 104]. Measurements by most groups did not include significant withdrawal periods, but Biggio and colleagues examined both chronic EtOH and withdrawal [105, 106]. Our results are described below.

5). The chronic intermittent ethanol (CIE) rodent model of alcohol dependence.

Twenty-five years ago, Kokka and Olsen established a rat model of the kindling hypothesis of alcohol dependence in humans [107, 108] and investigated the possible role of GABA_A_Rs [109]. In kindling, a sub-threshold stimulus such as an electrical stimulus or chemical convulsant drug is administered, and repeated with a defined duration, until the same stimulus produces a frank seizure on its own, and this supersensitivity is long-lasting. The kindling hypothesis of dependence development for CNS depressant drugs, including EtOH, sedative-hypnotics, and BZ addiction [110–112], was proposed based on the kindling of seizures.

## The CIE model and its relationship to human alcoholism

### CIE: 1991–2006

**Fig. 1:**
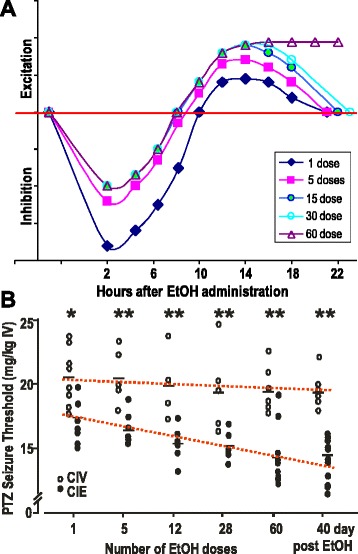
Time course of behavioral state and PTZ seizure threshold in rats given EtOH by gavage. **a**. Cartoon representation of behavioral state over time after administration of EtOH by oral intubation (gavage) in rat. EtOH exhibits maximum absorption into the brain by ~2 h, accompanied by behavioral depression. As the EtOH leaves the brain, activity (arbitrary units, amplitude depends on dose) returns to normal. Before the EtOH is even eliminated, the behavioral activity returns to normal and overshoots to produce a rebound hyperexcitability (withdrawal), then returns to normal by 24 h (blue diamonds). CIE after 5 doses (pink squares), reduces initial depression (tolerance) and slows return to normal with heightened severity of rebound hyperexcitability. After 60 doses (open triangles) in rats (30 in mice) the heightened withdrawal does not return to normal and stays elevated for at least 40–120 days, possibly for life [109]. This is the CIE ‘kindled’ state. **b**. Effect of CIE on PTZ seizure threshold: persistent decrease after cessation of EtOH treatment. EtOH, 5.0 g/kg/48 h, was given by oral intubation; PTZ seizure threshold was measured 18 h after EtOH. CIV rats tested at the same times as the CIE rats showed no significant changes in PTZ seizures. Horizontal bars indicate mean PTZ seizure threshold. ** *p* < 0.01. Reproduced from Kokka et al. (1993) [109] with permission. * *p* < 0.05

The rodent CIE regimen, with 5–6 g/kg EtOH administered to rats by gavage per day for at least 40 days (40–60 d) [111], was found to reduce the seizure threshold to the GABAergic convulsant drug pentylenetetrazol (PTZ, a GABA_A_R-chloride channel blocker), and this change lasted at least 40 d after EtOH was stopped (Fig. 1); importantly, the persistence of the changes (*kindling*) was dependent on the intermittent regimen, with repeated cyclical CNS depression and rebound hyperexcitable mini-withdrawal (Fig. 1a): providing an equivalent amount of EtOH continuously without repeated intermittent withdrawal produced a single serious withdrawal with seizures, but within a couple days there was no remaining effect on the animal, unlike with the CIE regimen (Fig. 1b). Other workers have demonstrated that the intermittent administration of EtOH, including periods of deprivation, can increase voluntary consumption [113, 114].

The chronic repetition of the mini-withdrawals leads to a persistent state of AWS in which the withdrawals become more severe and long-lasting, eventually becoming permanent. In other words, repetition turns a relatively normal brain activity involving plasticity into a pathological condition of uncontrolled hyperactivity. This is reminiscent of the kindling phenomenon in epilepsy research, in which seizures can be triggered by subconvulsant stimuli after they have been repeated over and over [115, 116]; eventually, seizures can become spontaneous, and once they do, they can occur for the rest of the person’s life. One facet of human alcohol dependence is increased seizure susceptibility, and delirium tremens and frank seizures are triggered by withdrawal from EtOH in very heavy abusers [117]. Greater susceptibility and/or severity of seizures is produced by greater periods of EtOH abuse and by previous withdrawals and/or withdrawal seizures. When the number of previous exposures and withdrawal episodes reaches a certain threshold, the severe withdrawal (AWS) [118] becomes persistent, possibly permanent. This led to the conclusion of a kindling-like phenomenon in human EtOH dependence [107, 112, 119, 120]. However, a significant reduction in seizure threshold can be measured during the mini-withdrawals experienced in rats after EtOH administration [109, 121]. This suggests that seizure susceptibility is, first, an integral component of withdrawal. Second, the increased severity and persistence of seizure susceptibility are signs of and critical ingredients of alcohol dependence. Numerous animal models employ this kindling-like regimen of intermittent episodes of EtOH intoxication and withdrawal, termed *chronic intermittent ethanol* (CIE) [113, 122–125].

We showed that in CIE, GABA_A_R binding was not much affected throughout the brain but that GABA_A_R function, assessed with a neurochemical assay of GABA-stimulated ^**36**^Cl^−^ flux in brain slices, was impaired specifically in hippocampal formation, but not in inferior colliculus, several lobes of cortex, thalamus, striatum, or cerebellum. Using extracellular electrode recording in hippocampal slices in collaboration with Dr. Igor Spigelman, we demonstrated a parallel reduction in paired-pulse inhibition [126] that was consistent with the increase in behavioral seizure susceptibility. Veatch and Gonzalez [127] presented similar evidence that intermittent EtOH with multiple withdrawals led to elevated excitability specifically in hippocampus, as detected by electroencephalography (EEG). We have further shown small changes in BZ modulation of GABA_A_R radioligand binding accompanied by a significant elevation in the GABA_A_R α4 subunit mRNA assessed by in situ hybridization histochemistry; the increase was relatively larger in hippocampus than in thalamus, despite higher levels of the subunit in thalamus [128]. This is consistent with elevated BZ-insensitive GABA_A_R and behavioral and cellular tolerance to BZ. Indeed, with intracellular sharp electrode recordings in hippocampal slices, we showed a reduction in allosteric modulation of GABA_A_R-mediated postsynaptic potentials by BZ and steroids but not by EtOH. EtOH enhancement of evoked synaptic potentials was, if anything, increased [126, 129]. In situ hybridization and reverse transcriptase-polymerase chain reaction (RT-PCR) revealed several changes in GABA_A_R subunits in CIE rat brain, including elevated γ2S in hippocampus and increased binding of the imidazo-benzodiazepine radioligand [^3^H]Ro15–4513 to diazepam-insensitive sites in cerebellum and forebrain, considered to involve the α6 and α4 subunits, respectively; we also showed GABA_A_R subunit mRNA level changes consistent with altered expression [130].

**Fig. 2:**
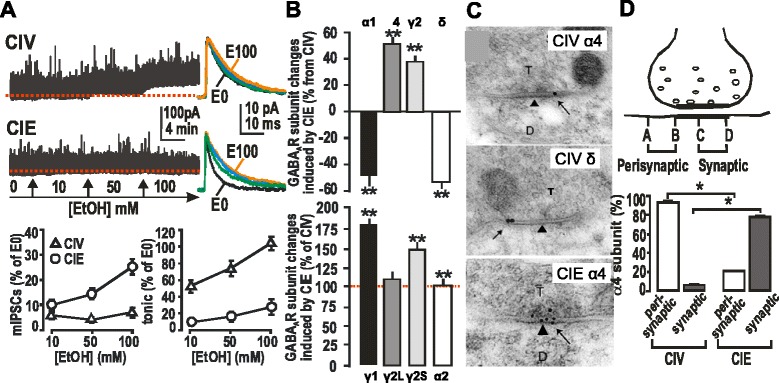
Plastic changes in GABA_A_R subunits and currents in rat hippocampal formation induced by CIE. A. EtOH-enhanced mIPSCs observed in hippocampal slices from CIE vs. CIV. Top left of A, recordings from CIV and CIE, including exposure to various concentrations of EtOH in the recording chamber. Top right of **a**, averaged mIPSC from each period response to EtOH applications during the recordings (left of **a**). Bottom of **a**, Summary of mIPSC area and tonic current for EtOH vs. pre-EtOH application. Redrawn from Liang et al., [81]. **b**. Upper: Summary of Western blot analyses of hippocampal GABA_A_R subunit peptides after CIE compared with CIV. Data are presented as percent changes from control peptide levels mean ± SEM. (*n* = 10 ~ 12 rats). ** *p* < 0.01, *t*-test. **b** Lower: GABA_A_R subunit mRNA levels assayed by PCR, normalized to the unchanged reference gene GADPH. Data are expressed as percentage of CIV group (control) mean ± SEM, ** *p* < 0.01, *t*-test. **c**. Post-embedding immunogold labeling reveals a change in α4 but not in δ subunit location from perisynaptic to synaptic sites in the molecular layer of the DG after CIE. In CIV (top and middle of **c**), colloidal gold labeling of the α4 subunit (arrows) was present on or near the plasma membrane of dendrites that contacted axon terminals (T). Gold particles were found predominantly at the outer edges of symmetric synapses (arrows) but not at the center of these synapses (arrowheads). After CIE (bottom of **c**), labeling for α4 was found mainly in the center of symmetric synapses (arrows). **d**. Quantitative analysis showed that perisynaptic labeling was found at 93% of α4-labeled synapses (open bar) in CIV (*n* = 3). In CIE (*n* = 3), perisynaptic labeling was observed at 22% (open bar) of labeled synapses, but synaptic labeling was evident at 78% of labeled synapses (black bar). * *p* < 0.001 vs. CIV. In contrast to the α4 labeling, δ subunit labeling (arrow) in CIE was present at perisynaptic locations but not within the synaptic contact (arrowhead). Figs. **a**, **c**, and **d** are reproduced from Liang et al. [81] with permission. Figs. **b** are redrawn from Cagetti et al. [131]

EtOH exposure causes changes in rodent brain GABA_A_R subunit composition and function, playing a crucial role in EtOH withdrawal symptoms and dependence. We showed [81, 131, 132] that CIE treatment and withdrawal results in decreased EtOH-enhanced δ subunit-containing GABA_A_R-mediated extrasynaptic current (Fig. 2a) correlated to down-regulated δ subunit (Fig. 2b). This is accompanied by increased EtOH sensitivity of GABA_A_R miniature postsynaptic currents (mIPSCs, Fig. 2a) correlated with hippocampal α4βγ2 subtypes including up-regulated α4 (Fig. 2b), and synaptic location demonstrated by post-embedding immunogold labeling electron microscopy (Fig. 2c-d).

Using subunit-specific antibodies, we measured GABA_A_R subunits by Western blotting in CIE rat hippocampus and demonstrated significant, persistent elevation in the α4 and γ2 subunits with a decrease in α1 and δ—in other words, a net “subunit switch” of α1 to α4 and δ to γ2. Using reverse transcription polymerase chain reaction (RT-PCR) assays, we found that CIE led to elevated mRNA levels for γ2S but not γ2L, as well as γ1 subunit but not α2; CIE-treated animals were shown to exhibit increased anxiety in the elevated plus maze assay, and behavioral tolerance to the sedative action of EtOH, BZ, and neurosteroids [131]. Steroids and BZ showed reduced enhancement of GABA_A_R synaptic and tonic inhibitory currents in hippocampal neurons recorded by patch-clamp electrodes in slices from CIE rats [132].

The changes found after CIE treatment did not appear to involve any gross pathology in either brain or liver [126]. Microscopic examination of tissue sections revealed no evident changes in the morphology and location of GABA-synthesizing neurons in hippocampus, thalamus, or neocortex [128]. Unbiased stereological cell counts in the nucleus accumbens of NeuN-stained sections showed no differences between CIE, single-dose EtOH, and vehicle-treated animals (I Spigelman, N Ahmad, J Liang, and RW Olsen, unpublished). This result is not consistent with evidence that exposure to a single very high dose of EtOH with blood levels of over 300 mg/dL, as experienced in human binge drinking, or to a very high level of cumulative alcohol exposure, as in human chronic alcohol abuse, produced significant neuronal cell death [133, 134]. We found no evidence for a significant increase in newborn neurons or for stem cell death in dentate gyrus (DG) of CIE rats versus normal controls (I Spigelman, J Liang, RW Olsen, and F Crews, unpublished). Thus, in our hands, high blood levels of EtOH administered by gavage, exceeding 250 mg/dL for several hours but not exceeding 275 mg/dL [65] were insufficient or too brief to produce the damage reported by other extreme exposures to EtOH. Nevertheless, CIE treatment is definitely a severe, abnormal stress to the brain.

CIE rats exhibit impaired hippocampal-specific spatial learning deficits [135], possibly due to decreased levels of neurosteroids. Neurosteroids (endogenous neuroactive steroids acting as GABA_A_R-PAMs: Smith [58]) may be increased by acute EtOH and decreased by chronic EtOH [136, 137], and thus could participate in GABA_A_R plastic changes induced by EtOH [59, 138]. CIE rodents have not been observed to exhibit spontaneous seizures but this has not been studied with sufficient care to conclude that there are none.

### CIE: 2007–2017

**Fig. 3:**
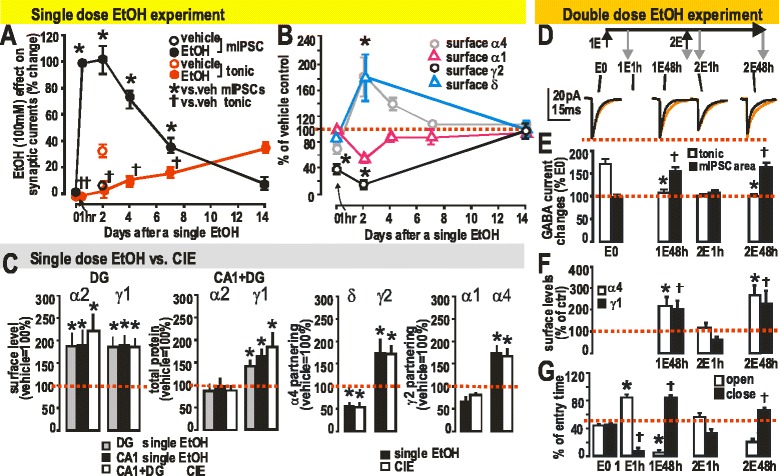
EtOH-induced plasticity of GABA_A_R subunits and currents in rat after single-dose EtOH, CIE, and two-pulse EtOH. **a**: Summary of changes in mIPSCs, and **b**: inhibitory tonic currents after single-dose EtOH vs. pre-EtOH application (redrawn from Liang et al. [65]). A single dose EtOH induces loss of EtOH-sensitive tonic current and gain of EtOH-sensitive mIPSCs. Mean ± SEM are shown as % of vehicle-treated controls (red dashed line, *n* = 4–6. * *p* < 0.05). **c**: Biochemical analysis of GABA_A_R subunit plasticity in rat DG within 24 h after single-dose EtOH compared with the changes induced by CIE, 40-d withdrawal. Surface protein levels of GABA_A_R subunits measured using protein cross-linking and Western blotting. Mean ± SEM as % of vehicle-treated controls (red dashed line, n = 4–6. * *p* < 0.05). The α2 and γ1 subunits cell surface expression are up-regulated by both one-dose EtOH and CIE, γ1 total peptide is up-regulated, but not α2; and the heteropentameric subunit partnerships up-regulated are α4βγ2 and α2β1γ1. **d**, Upper panel: The protocol of double-dose EtOH experiment. **d**, Lower panel: Averaged mIPSC from each time point response to EtOH applications during the recordings. **e**: Summary of acute EtOH-induced changes in tonic current and mIPSCs (*n* = 5). **f**: Quantification of surface levels of GABA_A_R (n = 4–6) by Western blots for GABA_A_R α4 and γ1 after cross-linking in slices. **g**: Anxiety assayed by EPM (*n* = 6). The duration time rats stayed in arms (% of total 5 min). **e**,**f**,**g**: all bars are compared to the control (E0 value for that parameter): * *p* , 0.05; † *p* < 0.05. In **e**, the control level (dashed red line, at 100%) applies only to mIPSCs; in **f**, the red line refers to control (100%) for both subunits; in **g**, the dashed red line corresponds to the E0 point for either open or closed arms. **c**,**d**,**e**,**f**,**g**: from Lindemeyer et al., [30] with permission

With the observations of remarkable GABA_A_R plasticity induced by CIE, we attempted to learn molecular mechanisms and functional relevance through studies to determine the minimum dose, duration, and frequency of EtOH administration required to produce the changes. We found that a single high, intoxicating, dose of EtOH administered by gavage was able to induce many of the same changes in behavior, GABA_A_R subunit composition, and hippocampal neuron pharmacology seen in CIE, but the changes were transient [65]. Thus, we showed that within 1 h the α4 and δ subunits, but not the α1 or γ2 subunits, were reduced at the cell surface, accompanied by loss of EtOH enhancement of tonic inhibitory currents but no change in synaptic pharmacology. Thus, the first target of EtOH action, the extrasynaptic δ subunit-containing GABA_A_Rs [68] are the first to respond with plastic changes. After 24 h but not at 1 h, one could detect increased cell surface and increased total levels of γ2 and α4 subunits, decreased levels of α1 subunit, and a tolerance to BZ enhancement of both extrasynaptic and synaptic currents (Fig. 3a, b). These changes are probably the result of altered gene expression; they may be triggered somehow by the reduced tonic inhibition or even the reduced synaptic inhibition seen at several hours post-EtOH. Altered protein synthesis may also be initiated by the EtOH exposure itself, but requires a longer time to reach experimental detectability. At 12 ~ 24 h, the animals exhibited tolerance to BZ- and high dose EtOH-induced loss of righting reflex (LORR), and the synaptic currents became more sensitive to EtOH (as in CIE), but they returned to normal within a few days. This included the δ subunit remaining low for 1 ~ 2 days and then returned to normal [65]. All the changes require the CIE regimen to become more persistent, fortunately for human alcohol users, who have the option to refrain from chronic use. Failure to do so is called AUD.

GABA_A_R plasticity induced by CIE demonstrated a correlation between the degree of tolerance induced for a series of GABAergic sedative-hypnotic drugs to produce LORR and the degree of tolerance induced for the same drugs to enhance GABA_A_R-mediated tonic inhibitory currents in hippocampal neurons [139]. On the other hand, the anticonvulsant and anxiolytic actions of GABA_A_R PAMs (EtOH, neurosteroids, propofol, barbiturates, as well as the GABA analogue gaboxadol show little tolerance [81, 131, 132, 135, 139]).

However, the CIE rodents and the one-dose EtOH-treated animals show elevated sensitivity of GABA_A_R-mediated mIPSCs to modulation by low mM EtOH in the recording chamber [81]. In CIE we observed an increase in α4βγ2 GABA_A_Rs, including movement of the α4 into the postsynaptic membrane. The δ subunit was not elevated and did not accumulate in the synaptic membrane, and the increased EtOH modulation of mIPSCs was also observed in the alcohol- naïve knockout (KO) mouse for both GABA_A_R α4 subunit [140] and δ subunit (J Liang, RW Olsen, and I Spigelman (2002), unpublished) and might account for the lack of reduction in many EtOH behaviors in these mice [141–143]. Furthermore, we posited that these EtOH-sensitive GABA_A_Rs are apparently up-regulated by EtOH treatment, and might be positioned in brain locations where they might mediate the continued EtOH-sensitivity in regions needed for the *positive reinforcement reward as well as anxiolytic efficacy of EtOH in the dependent individual, animal or human*. Therefore, we asked: what might be the subunit composition of GABA_A_Rs accounting for this increased sensitivity to EtOH of mIPSCs? Below we describe our discovery of a GABA_A_R subtype that meets these requirements.

We have also extended the CIE model to the mouse [111]; the mouse required a slightly modified regimen of EtOH administration due to higher metabolism, but we managed to achieve similar EtOH-induced GABA_A_R plasticity as in rats. The goal is to establish a short term intermittent EtOH (SIE) mouse model to replicate the information found in the established chronic intermittent EtOH (CIE) model. In comparison to the CIE model, the SIE mouse model can be more easily combined with genetic technology for in depth studies of the underlying mechanisms of alcoholism. C57Bl/6 mice were separated into short intermittent vehicle (SIV) and SIE groups. SIV and SIE mice were gavaged drinking water or ethanol respectively, every other day for five doses and from day 11th, once a day for 30-d. SIV mice served as the control group. We evaluated behavioral changes after two day and 40-d withdrawal from SIE and compared with CIE. The results are consistent with previous reports and indicate that SIE mice, like CIE rats, have greater anxiety, hyperexcitability, and tolerance to acute EtOH-induced LORR than SIV [111]. Then we analyzed genetically engineered animals with GABA_A_R subunits knocked out, in, or down [30, 140, 141, 144, 145]. The α4KO mouse showed reduced GABA_A_R-mediated tonic inhibition throughout the brain and reduced electrophysiological and behavioral effects of gaboxadol [144], including also reduced modulation of tonic currents by low mM EtOH [140], but, disappointingly, normal behavioral responses to EtOH [141, 142]. We did establish in preliminary evaluation that the α4KO mice showed a *blunted* effect of CIE treatment, especially elimination of tolerance development to the intoxicating effects of EtOH, including sedative-hypnotic, motor-incoordinating effects [146]. We extended the model to primary cultured hippocampal neurons [147], where certain variables could be more closely controlled than in the animal. Exposure of the cultured neurons (cultured at embryonic age 18-d, and studied at ≥15-d in vitro (DIV), but not earlier, at which time, they exhibited both δ subunit expression and EtOH-enhanced tonic inhibitory currents, showed a rapid down-regulation of EtOH-enhanced tonic inhibitory currents as well as down-regulation of δ subunit, mimicking the EtOH effect in vivo [147].

Both covalent biotinylation of cell surface proteins (technique most suitable for cultured monodisperse cells) and crosslinking of cell surface proteins, to exclude them from the SDS gel during electrophoresis (technique most suitable for brain slices) that the rapid, within hours, and likely minutes, down-regulation of α4βδ GABA_A_Rs by EtOH exposure involves protein internalization (endocytosis). In the case of δ subunit, this is clathrin-dependent [71]. This is consistent, as described above, with the extrasynaptic δ subunit-containing GABA_A_R as early responders to low millimolar EtOH, and likely requires a conformational change in the intracellular domain of δ to allow it to bind the clathrin accessory subunit when the GABA_A_R protein binds GABA ‘too long,’ as when the GABA concentration is prolonged at a high concentration, or by the presence of a PAM like EtOH to enhance GABA binding. Terunuma et al. [56] showed that during status epilepticus, presumed massive synaptic GABA release and binding to synaptic (α1, 2,and 3) subunit-containing GABA_A_R molecules exhibit internalization triggered by the prolonged activated protein conformation with the β3 subunit becoming a substrate for a phosphatase that removes phosphate and allowing endocytosis. This mechanism was ruled out for the δ-containing GABA_A_R [71]. The down-regulation of δ-GABA_A_Rs reverts to normal after some hours to days of EtOH removal but fails to normalize after multi-dose CIE regimen [65, 81]. We have argued that this is probably not due to cell death or damage. One possibility under consideration is the possible loss of a δ membrane surface location-stabilizing protein factor, either the fragile X protein FMRX or another protein exhibiting increased translation regulated by FMRX. Mice lacking FMRX were found to lose cell surface GABA_A_R δ subunit without change in total δ protein [148].

Although most measurements were made, justified by region-specific changes in GABA_A_R pharmacology and expression related to EtOH action, in the hippocampal formation, changes in EtOH-sensitive GABA_A_R s throughout the CNS are likely (basolateral amygdala: [149–151]; ventral tegmental area: [152], nucleus accumbens: [153]), thus affecting many behaviors. These would be expected to show regional and cell type specificity if they depend on the presence of the GABA_A_R subtypes that we found are down-regulated (δ, α1) or up-regulated (α4, α2) by EtOH exposure. We suggest that the plastic changes in hippocampus are a model for changes in other regions and these could well involve areas/circuits critical for both the dopamine reward system (ventral striatum/nucleus accumbens and ventral tegmental area), and for maintained anxiolysis (amygdala, hippocampus) in the EtOH-dependent individual, rodent or human.

### The latest news on the CIE rodent model of AUD

**Fig. 4:**
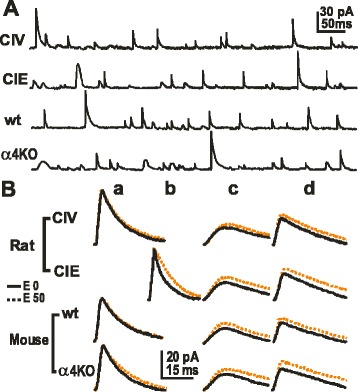
Hippocampal cells mIPSC kinetics patterns for GABA_A_R subtypes in CIE rats and α4KO mice. **A**: mIPSC sample traces of CIE- vs. CIV-treated rats and α4KO and WT mice in hippocampal DG cells. **B**: Averaged mIPSC shape patterns detected by DataView revealed 3–4 relatively abundant distinct templates. In CIV, mIPSC patterns ‘a’, ‘c’, and ‘d’ were detected. Pattern ‘a’ is a standard shape, typical rise and decay kinetics; patterns ‘c’ and ‘c’ are slow rise-slow decay patterns correlated in abundance (not shown here) with α2 subunit subtypes. Three patterns of mIPSCs were also detected in CIE, but the ‘a’ pattern was not seen in CIE, and replaced by the slower decay pattern ‘b’. See text for interpretation that ‘a’ is mainly α1 and ‘b’ is mainly α4 subunit subtypes (as in Liang et al., 2006). Patterns of mIPSCs in WT and α4KO mice are similar to CIV rats, with peaks ‘a’, ‘c’, and ‘d’. However, the abundance of pattern ‘d’ was increased in CIE relative to CIV and in the α4ko mouse relative to WT. Since the CIE but not CIV, and a4KO mouse but not WT exhibited EtOH-enhanced mIPSCs, we examined recordings of these four animal groups with 50 mM EtOH (E50, dashed line) compared to without EtOH (E0, solid line) in the recording chamber. Peak pattern ‘a’ was not significantly enhanced by EtOH, but ‘b’, ‘c’, and ‘d’ were enhanced. Peak ‘b’ in CIE correlates with up-regulated α4, and is not seen in the α4KO mouse. Peak ‘d’ is up-regulated in both CIE rat and α4KO mouse, as is the α2 subunit surface expression, and peak ‘d’ has slow kinetics consistent with the α2 subunit subtypes. Its increase in abundance correlates with the increased average stimulation by EtOH in the recording chamber for both CIE and α4KO. Reproduced from Lindemeyer et al. [30] with permission

Acute and chronic EtOH intoxication in rats increased surface levels of GABA_A_R α2 and γ1 subunit protein in hippocampus, using cross-linking and Western blots*.* CIE and single-dose EtOH administration up-regulate GABA_A_Rs composed of α2β1γ1 subunits that bind to gephyrin, demonstrated by co-immunoprecipitation (co-IP) experiments [30]. In order to determine which subunits partner with γ1, both γ1 and γ2 co-IP (positive control) Western blots were probed for α1, α2*,* α4, and α5. In contrast to γ2, which was found to associate with different α subunits, γ1 primarily co-assembled with the α2 subunit (Fig. 3c). The γ1 antibody did not co-IP γ2 and vice versa. The preferred β subunit partner for the α2γ1-containing GABA_A_Rs was identified by co-IPs with β1-, β2-, or β3-specific antibodies, probing for γ1 and γ2. The γ1 preferentially formed a receptor complex with the β1 subunit, with a small extent with β3 and no β2. By contrast, the γ2 equally partnered with β1 and β3 and somewhat less with β2. These data identify GABA_A_Rs composed of α2, β1, and γ1 subunits in hippocampal CA1 and DG regions that are found to be up-regulated after CIE and single-dose EtOH exposure (Fig. 4). The selective partnering of γ1 with α2 allows use of γ1 as a marker for the up-regulated pool of cell surface α2 subunits (a minor subtype of α2, which preferentially partners with γ2). Western blotting with a gephyrin antibody suggests at least some postsynaptic localization of γ1-containing receptors at inhibitory synapses. We also showed by co-IP studies on solubilized membrane proteins from hippocampus of CIE-treated rats that the previously reported [81] up-regulated α4 and γ2 subunits and down-regulated α1 and δ subunits are accompanied by a net switch in partnering of α4 from δ to γ2 and partnering of γ2 from α1 to α4; the new α2 is selectively partnered with γ1, β1, and gephyrin [30]. This demonstrates that the up-regulated GABA_A_R subtypes are α4βγ2 and α1β1γ1. These up-regulated subtypes are probably satisfactory for replacing the lost synaptic and extrasynaptic inhibitory currents normally mediated by the EtOH-induced GABA_A_R subtypes [67].

**Fig. 5:**
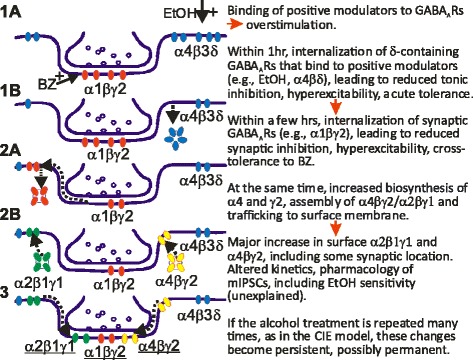
A Reasonable Hypothesis of GABA_A_R Subunit Plasticity Induced Within Two Days by One Dose of EtOH. Administration of EtOH to rats leads to changes of physio-pharmacological properties in GABAergic ionotropic receptor-mediated inhibitory synaptic transmission in hippocampus. The text at right of figure explains the time course of EtOH-induced plasticity, and how these same changes become persistent after CIE treatment. Reproduced from Lindemeyer et al. [30] with permission

Time-dependent changes of α4- and α2γ1-containing GABA_A_R subtypes are tightly correlated with up- and down-regulation of EtOH-sensitive mIPSCs and withdrawal anxiety following one or two doses of EtOH. The α2β1γ1 and α4βγ2 receptor subtypes have a similar, not easily distinguishable pharmacological profile so we could not unambiguously distinguish them based on pharmacology. In order to understand better the process of CIE-induced synaptic restructuring, we studied effects on rats given a single dose and double dose of EtOH (Fig. 3d, e, f, g). Animals gavaged with a single-dose EtOH (5 g/kg) repeated at 48 h, show within 1 ~ 2 h a parallel loss of α4 and γ1 (marker for α2), loss of EtOH-enhanced mIPSCs in hippocampal slice patch-clamp recordings, and loss of withdrawal signs seen at 48 h after the 1st dose of EtOH (tolerance to EtOH- and BZ-LORR; increased anxiety using the elevated plus maze (EPM) technique, and sensitivity to PTZ seizures). Testing again at 48 h after the 2nd EtOH dose showed return in parallel of all the above: anxiety, EtOH-sensitive mIPSCs, and the up-regulated α2 and α4 (Fig. 3d, e, f, g). Thus either the α2 or α4 might mediate the EtOH-sensitive mIPSCs. Forty-eight hour after the 1st EtOH dose (“one-dose”), the changes already described are seen (behavioral withdrawal, including tolerance to EtOH and BZ sedation and LORR; increased hyperactivity including increased sensitivity to PTZ seizures; and increased anxiety in EPM; loss of EtOH-enhanced tonic inhibitory GABA_A_R currents, but appearance of EtOH-enhanced mIPSCs; and down-regulation of δ and α1 and beginning of up-regulation of α4βγ2 GABA_A_R. Now a 2nd EtOH is administered. Within 1 ~ 2 h, the EtOH-sensitive mIPSCs are gone; the elevated surface α4 and γ1 (marker for the subset of α2-GABA_A_R, α2β1γ1 subtype) are gone; and the withdrawal anxiety is reduced. At 48 h after the 2nd EtOH, all these parameters return to the levels seen 48 h after the 1st EtOH dose: EtOH-sensitive mIPSCs are present; α4 and γ1(α2) subunits are present; withdrawal anxiety is present (Fig. 5). Thus both the α4- and α2-containing GABA_A_R subtypes are changing rapidly up and down after EtOH, one or two doses, and this approach cannot distinguish which might be more important for EtOH-sensitive mIPSCs; possibly both are important [30].

CIE induces up-regulation of one or more GABA_A_R subtypes with slow mIPSC decay kinetics. To better understand how changes in subunit combinations alter GABA_A_R function and responsiveness to acute EtOH, we measured mIPSCs in DGCs from hippocampal slices of CIV (control) and CIE-treated rats (Fig. 4A), and analysed them for shape patterns using the optimally scaled template method [154] implemented in DataView software to identify kinetic patterns of mIPSCs (e.g., fast rise and fast decay, slow rise and slow decay). Then, we used these identified patterns as templates to detect differently shaped mIPSCs in the recording traces. An acceptable error level was set which is the degree of similarity an event must have to the templates to be included in the search results. We observed that mIPSCs exhibited a few relatively consistent waveform patterns in the recordings. The detected mIPSC peak patterns were averaged, mIPSC patterns were classified (Fig. 4B, a, b, c, and d), and their kinetic decay constants τ were determined, and % abundance of each template was counted (not shown in figure presented) in a sufficiently large epoch of recordings as in Fig. 4A.

We reasoned that different GABA_A_R subtypes have been claimed to be recognizable as different subunit-containing subtypes based on mIPSC kinetics. Different α subunit-containing native or recombinant GABA_A_Rs with αβγ2 [155–157] could be distinguished from each other, α1 faster than α2, and can be detected in neurons by the peak-shapes of their mIPSCs, which provide a ‘fingerprint’ for individual α subunits including α2. Recombinant α4β2γ2 have accelerated deactivation compared to their α1 or α5 counterparts, correlating with up-regulated α4 subunit in a hyperexcitable model examining hippocampal slices in a neurosteroid-withdrawn rat [158]. Also, the γ1 subunit-containing receptors (especially with α2) exhibit slower activation and deactivation rates than the respective γ2-containing GABA_A_Rs expressed in engineered synapses [159]. mIPSC rise time is sensitive to multiple physical variables of synaptic transmission other than receptor subunit composition [160] However, the decay time is less sensitive to these variables as they are rather random, but more sensitive to the nature of the postsynaptic receptor-channels, such as rates of channel closing and agonist dissociation [161] mIPSC shape is highly sensitive to synaptically released peak GABA concentrations and durations [162], but [quote], “differential expression of GABA_A_R α subtypes with either a variable or constant ratio from synapse-to-synapse and cell-to-cell, allows them to fulfil individual cellular requirements in network dynamics” [163].

CIV animals exhibited three distinct mIPSC waveform patterns (Fig. 4B): one standard pattern (‘a’, abundance ~48%), and the other two both display a slower decay pattern (‘c’, ~37%; and ‘d’, ~16%). CIE animals likewise showed three distinct mIPSC shape patterns, but one was changed: a ‘fast’ decay pattern (peak pattern ‘b’, ~42%); and two apparently similar to CIV patterns with a slow decay (peak pattern ‘c’, ~22%); and a very slow decay pattern (peak pattern ‘d’, ~36%). The standard peak pattern ‘a’ seen in CIV had disappeared in CIE, whereas the ratio of ‘c’ to ‘d’ had reversed, from ~2:1 (CIV) to ~2:3 (CIE). Also, importantly, pattern ‘d’ had clearly increased in abundance, while ‘c’ may have decreased [30].

To better understand the different pattern of peaks possibly carried by particular GABA_A_R subtypes, we extended this analysis to genetically engineered α4KO mice (Fig. 4B). The patterns of mIPSCs in WT mice, untreated. ‘a’, abundance 46%; ‘c’, abundance 36%; ‘d’, 18% are similar to CIV rats, while α4KO mice show mIPSC waveform patterns ‘a’ (abundance ~36%), ‘c’ (~35%), with increased abundance of ‘d’ (~29%) (Fig. 4B [abundance not shown in figure]).

EtOH (50 mM) perfused into the recording chamber potentiated mIPSCs by prolonging decay time and/or increasing charge transfer (area under the curve), as previously observed Liang et al., [81]. For CIE rats, we therefore examined whether EtOH (50 mM) application enhanced the current of the various types of mIPSCs detected (Fig. 4B). We found that acute EtOH potentiated some specific GABA_A_R mIPSCs. The area of the mIPSCs increased greatly in CIE pattern ‘d’ with EtOH in the recording chamber (Fig. 4B), as did its abundance as a fraction of total mIPSCs in the recording trace. In vitro sensitivity to EtOH modulation correlated in time with the up- and down-regulation of the α4- and especially the α2-containing GABA_A_R subtype species (Fig. 4B). The mIPSC peak pattern ‘a’ was previously [81] correlated with the down-regulated α1 subtypes, and the peak pattern ‘b’ was correlated to the CIE-up-regulated synaptic α4-subtype. But what GABA_A_R subtypes account for peaks ‘c’ and ‘d’? These cells also contain α2- and α5-GABA_A_R subtypes. The α2 are considered synaptic and the α5 primarily extrasynaptic [164, 165]. Peak ‘d’ is almost certainly an up-regulated α2 subtype. To summarize, two novel GABA_A_R subtypes are up-regulated after acute EtOH treatment and CIE. Cell surface levels of both subtypes are tightly synchronized over one- or two-dose EtOH administration with changes in anxiety behavior and the abundance of EtOH-enhanced mIPSCs. We directly related changes in surface expression of GABA_A_R subunits (down-regulation of α1 and δ, up-regulation of α4, α2, γ1, and γ2) with a decrease in heteropentameric extrasynaptic α4βδ- and synaptic α1βγ2-containing GABA_A_Rs and an increase in postsynaptic α4βγ2- and α2β1γ1-containing GABA_A_Rs in hippocampal neurons (Fig. 5).

Up-regulated α2 subtypes correlated with the appearance of synaptic currents enhanced by EtOH (>10 mM). EtOH-enhanced mIPSCs have also been observed in untreated α4KO mice [140, 145], in which the EtOH-sensitive subtype cannot contain α4. The α2 subunit is co-localized with gephyrin and presynaptic glutamic acid decarboxylase (GAD) at both DGC cell bodies and axon initial segments [163] and is up-regulated in the hippocampus of α4KO mice [140, 145]. The decrease in α1βγ2- and gain of α4βγ2- and α2β1γ1-containing GABA_A_Rs change the kinetics and pharmacological properties of mIPSCs. We previously found decreased diazepam or zolpidem enhancement of mIPSC decay constants and a markedly increased area by the imidazobenzodiazepine partial inverse agonist Ro15–4513 in hippocampal slices after CIE [65, 81, 131, 132, 139], and single-dose treatment in vivo [65]. These pharmacological and subunit changes were reproduced in primary cultured embryonic hippocampal neurons after 15-d in vitro, 24 h after exposure for 30 min to EtOH (50 mM) [147].

Fig. 5 shows a reasonable hypothesis of GABA_A_R plasticity induced by EtOH in rat hippocampus (updated from Liang et al., [65]). This shows how synaptic and extrasynaptic GABA_A_R subtypes change rapidly in surface expression after in vivo exposure to EtOH and that the plastic changes become persistent after CIE treatment. Note that in this simplified cartoon we have grouped all the game players in a single synapse, which is not likely to be the actual situation.

### AUD as an aberrant plasticity phenomenon of GABA_A_Rs in brain [67]

EtOH induces down-regulation of the first responder receptors, which produces acute tolerance to EtOH, and also triggers the loss of additional GABA_A_R subtypes resulting in hyper-excitability. Adaptations to correct this change do restore inhibition, but it is abnormal, and the animals remain hyperexcitable. Although the EtOH-sedating GABA_A_R are gone, the replacement GABA_A_Rs exhibit EtOH-enhanced synaptic GABA_A_R inhibitory currents [81]. The CIE-treated rats that show ‘kindling’ to the GABA_A_R channel blocking convulsant drug PTZ-induced seizures [109], and increased anxiety [131], and tolerance to sedative-hypnotic effects produced by EtOH, BZ, and all GABAergic sleep aids (and likely drug-resistant insomnia in man) [139], do not exhibit tolerance to the anxiolytic action of EtOH in the dependent CIE rats [81] and presumably in dependent humans. We posit that the retained sensitivity to the anxiolytic effects of EtOH is important to development of withdrawal-promoted drinking. The hallmark of alcohol addiction is increased drinking and this has been demonstrated by many to result from CIE treatment in rodents [113, 124]. All these behavioral features of alcohol addiction are persistent for 4 ~ 12 months, and probably for life [67, 109]. We have learned that the new EtOH-enhanced synaptic GABA_A_R in CIE are the up-regulated α4βγ2 and, especially, α2β1γ1.

The behavioral changes of AWS can be explained by persistently reduced GABA_A_R-mediated inhibition due to EtOH-induced plasticity of GABA_A_Rs. When this becomes persistent due to the CIE treatment, this can be termed ‘aberrant plasticity’ [109]. The receptors for the very important rapid neurotransmitters glutamate, and especially GABA, are liable to aberrant plasticity and in a position to do the most harm [166]. In the case of CIE, the treated individual has all the signs of AWS which is an extreme hyperexcitable condition, contributory to increased EtOH consumption. Anxiety (feeling stressed), insomnia, and increased seizure susceptibility (kindling?), also aspects of AWS, would seem to be critical aspects of dependence development [67, 107, 120, 167]. However, we do not know what additional factors, including susceptibility genes, if any, are required to generate actual addiction (alcoholism).

## Conclusions, discussion, speculation

### Remaining questions about the rodent CIE model

The CIE animal model exhibits EtOH-induced plastic changes in GABA_A_R subunit composition and localization. Acute EtOH induces transient changes in a prescribed temporal sequence, starting with decreased extrasynaptic α4βδ, followed by decreased synaptic α1βγ2 detectable within hours but possibly triggered earlier; about the same time a detectable increase in α4βγ2, including surface expression and synaptic localization, is observed in hippocampus [65] and nucleus accumbens [153], as well as increased synaptic α2βγ, primarily α2β1γ1-gephyrin in hippocampal formation [30] and basolateral amygdala (BLA) [150]. The major question remaining is, ‘*How do these changes become persistent* after EtOH administration that produces a certain number (30~60) of cycles of behavioral depression and hyperexcitable rebound mini-withdrawals?’

Attempts to answer this question have included more detailed analysis of the nature and time course of changes in the subunit composition, both total and surface expression, as well as subtype subunit partnering measured by co-immunoprecipitation and Western blotting, including receptor-associated proteins, in hippocampal formation or microdissected DG or CA1. This has been correlated with alcohol intoxication and withdrawal behaviors and patch clamp recordings of GABA_A_R currents in hippocampal slices to determine channel amplitudes, kinetics, and pharmacology, including sensitivity to modulation by EtOH applied in the recording chamber. We have also extended the CIE model to the mouse, and analyzed genetically engineered animals with GABA_A_R subunits knocked out, in, or down [30, 140, 141, 144, 145]. Also, we extended the model to primary cultured hippocampal neurons [147], where certain variables could be more closely controlled than in the animal. To determine the nature of the changes more precisely, we attempted to pinpoint the regulated step(s) to aspects of protein cell biology: transcription, translation, assembly, and trafficking, including membrane surface expression? We examined the possible role of associated proteins, protein phosphorylation and/or neurosteroids. In some cases, we attempted to determine whether all the changes seen were occurring in the same cells.

The rapid removal of α4βδ and somewhat slower build-up of α4βγ2 appears to involve de novo synthesis of α4 as well as assembly selectively of α4βγ2 and membrane insertion, plus synaptic localization [81], not normal for α4-GABA_A_R [168]. The regulation of α4 transcription has been demonstrated to involve up-regulation of immediate early gene transcription factors, like heat shock proteins elevated by EtOH exposure [169] and/or BDNF, elevated by seizures [170, 171], and/or by microRNAs, possibly suggesting epigenetic mechanisms [172]. We have speculated (below, also Lindemeyer et al., [31]) that the DGC GABA_A_R synapses after CIE treatment may be abnormal in some way to explain unusual physiology and pharmacology, such as mIPSC kinetics and sensitivity to low millimolar EtOH modulation. This could involve an associated protein, possibly gephyrin/ collybistin [169–171], or even PSD-95 (Lindemeyer AK, Liang J, Olsen RW (2013), unpublished), normally part of glutamate receptor synapses [43]. Once formed, these synapses might be for some reason resistant to turnover and/or reversion to the normal structures, perhaps due to aberrant matrix structure [173–175].

The α1-GABA_A_R expression and surface localization have been demonstrated to be regulated in vivo by a complex region- and cell-specific protein kinase A and protein kinase C system [53, 176]. In vitro studies in cultured neurons helped clarify the timing and interactions of the various phosphorylation events relevant to both gene expression and trafficking triggered by EtOH exposure [177, 178]. As mentioned above [145], we found in mice lacking the GABAAR α4 subunit that α1- and α2-GABA_A_Rs were prevalent contributors to the mIPSCs in DGC which were enhanced by EtOH in the recording chamber and were rapidly down-regulated by EtOH exposure, unlike in naïve wild type mice or rats, consistent with early internalization of early responder-GABA_A_Rs to EtOH in vivo. We also found that the α2-GABA_A_R subtype of synaptic current that is most abundantly up-regulated in cell surface expression after CIE and that is most sensitive to EtOH modulation in the recording chamber [30]. Since most of the up-regulated pool of α2 in this region and possibly elsewhere such as amygdala [150], is complexed in a heteromeric GABA_A_R with α2β1γ1 [30], a rare subtype in most regions, and this might produce synapses differing from ‘normal, including low turnover and persistent phenotype.

### Suggested importance of GABA_A_R plasticity in AUD and role of α2 subunit

**Fig. 6:**
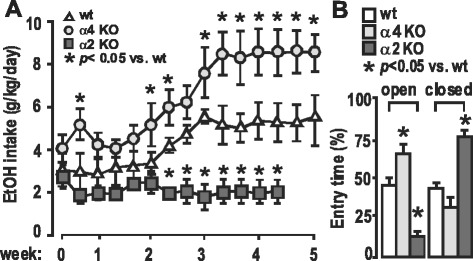
Two-Bottle Choice Assessment of EtOH Drinking by GABA_A_R Wild Type and α2KO and α4KO Mice. **a**. EtOH preference assayed by voluntary access to EtOH (15%) in the 2 BC. (Δ, WT [C57/BL/6]; Ο, α4KO (G Homanics); and **ם,** α2KO (U Rudolph), *n* = 6–8). After the 3rd week, the EtOH intake in the α4KO group became significantly higher than that in the WT group. In contrast, the α2KO group did not show EtOH preference. **b**. Anxiety assay after 3 weeks of 2 BC measured by EPM (*n* = 6 ~ 8). The α4KO EPM data show reduced anxiety relative to wild type, while the α2KO exhibit more anxiety

Investigating the relevant GABA_A_R subtypes for a causative role in CIE/AUD, we tested the U. Rudolph α2KO mouse [179] in the two-bottle choice paradigm to estimate voluntary EtOH consumption and found these animals to exhibit lower acceleration of drinking than wild type [180]. However, the α4KO mouse of Homanics [140] showed higher than wild type level drinking (Fig. 6). This α2KO result conflicts with results from both D. Stephens’ lab [181] and A. Harris’ lab [182]. Behavioral scientists will understand that variable results are routine in studying different strains of animals in different labs with slightly different methodology, so more study is needed to clarify this situation, but clearly the α2-GABA_A_R subtypes are candidates of interest in AUD. We mentioned above that evidence suggests that the α2 subunit-containing GABA_A_Rs participate functionally in critical neurocircuitry involved in the positive reinforcing effects of EtOH [27–30], as they are for BZ [32, 33], and other drugs of abuse [35]. We posit that the α2-GABA_A_Rs are needed for the development of EtOH dependence. Increased expression and function might be associated with dependence, and reduced expression and function somehow associated with less susceptibility to developing dependence. This is consistent with genetic association of GABRA2 with alcoholism [26].

Is the α2-GABA_A_R in critical brain regions and subcellular membrane locations critical for the anxiolytic response to EtOH and for the elevated drinking in the dependent CIE mouse? If so, it will establish insights into EtOH dependence and drinking and possible therapies that will set the stage for the next generation of questions to answer. For example, how does the EtOH-induced plasticity, namely induction of α2β1γ1 occur, and can it be prevented, or remedied, e.g., with subtype-selective drugs? How do the EtOH-induced plastic changes become persistent, and can that be prevented, or remedied? Is the switch at the level of gene expression or protein trafficking? Where do genetic differences, which we know affect human alcoholism, manifest in such an addiction model? Are the α2β1γ1 and α4 gene cluster SNPs really important? One factor that might be critical for addiction and individual differences is stress [183]: how important is it? Is it possible the GABA_A_R-enhancing (calming) neurosteroids (metabolites of progesterone and corticosterone) participate at this level? We are giving a lot of credit to GABA_A_R plasticity in certain circuits: what about GABA_A_R changes elsewhere and the behaviors affected (e.g., [28])? How about the glutamate receptor plasticity that has also been observed? How do those interact with GABA_A_R changes? It is likely that numerous neuropsychiatric disorders, not just drug abuse, involve aberrant receptor plasticity and this may be complicated by chronic therapy with negative or positive allosteric modulatory drugs (NAM or PAM) for the receptors involved. Successful therapy for AUD based on GABA_A_R plasticity would be impetus for more research in the receptor plasticity field.

Administration of any GABA_A_R-PAM drug, including EtOH, neurosteroids [58], benzodiazepines [60, 89], and anesthetics [61], can induce GABA_A_R down-regulation, compensatory plasticity, producing tolerance and withdrawal, as well as aberrant plasticity involving GABA_A_Rs and associated negative effects on behaviors. Neurosteroid GABA_A_R-PAMs have been demonstrated to produce a hyperexcitable model upon withdrawal, accompanied by anxiety, reduced GABA_A_R-mediated inhibition, and tolerance to BZs [58, 184], with many changes mirroring with minor differences those reviewed here for acute and chronic EtOH administration. It has also been suggested that neurosteroids (endogenous neuroactive steroids acting as GABA_A_R-PAMs [58] may a) actually mediate some (but clearly not all) pharmacological actions of EtOH [185]; b) may be increased by acute EtOH and decreased by chronic EtOH [136, 137] and thus participate in GABA_A_R plastic changes induced by EtOH [59, 138]; c) be particularly sensitive to sex (progesterone) and stress (deoxycorticosterone) endocrine status, since one- or two-step metabolites of the hormones are endogenous GABA_A_R PAM neurosteroids [58], and appear to change important brain functions during the menstrual cycle and play a neuropsychiatric role in premenstrual syndrome [186], puberty [187], pregnancy [188], post-partum depression [189], involving GABA_A_R plasticity, both by inducing changes in GABA_A_R expression and localization [98], and by modulating GABA_A_R sensitivity to EtOH [58, 190]. It is currently difficult to assess the importance and detailed role of neurosteroids in AUD involving GABA_A_Rs, but this remains an area of interest requiring more research.

## Abbreviations

AUD: Alcohol use disorder
AWS: Alcohol withdrawal syndrome
BZ: Benzodiazepine
CIE: Chronic intermittent ethanol
CIV: Chronic intermittent vehicle
DGC: Dentate gyrus cells
EPM: Elevated plus maze
EtOH: Ethanol
GABA_A_R: GABA_A_ receptor
LGIC: Ligand-gated ion channel
NAM: Negative allosteric modulator
NMDA: N-methyl-D-aspartate
PAM: Positive allosteric modulator
RT-PCR: Reverse transcription polymerase chain reaction
SIE: Short intermittent ethanol
SIV: Short intermittent vehicle
